# Taxonomic Description and Genomic Characterization of *Saccharibacillus soli* sp. nov., Isolated from Copper Mine Soil, Khetri, Rajasthan, India

**DOI:** 10.3390/microorganisms14051150

**Published:** 2026-05-19

**Authors:** Himani Darangwal, Bhawna Vyas, Munesh Kumari, Ojal Bansal, Shanmugam Mayilraj, Venkata Ramana Vemuluri

**Affiliations:** 1 Microbial Type Culture Collection & Gene Bank (MTCC), CSIR-Institute of Microbial Technology (IMTECH), Sector 39-A, Chandigarh 160036, India; himani.c1088@gmail.com (H.D.); vyasbhawna@gmail.com (B.V.); mayailrajs@bentoli.com (S.M.); 2 Department of Microbiology, Panjab University, South Campus, Sector-25, Chandigarh 160014, India; 3 Bentoli AgriNutrition India Pvt. Ltd., Anna Salai, Chennai 600002, India; 4 Academy of Scientific and Innovative Research (AcSIR), Gaziabad 201002, India

**Keywords:** copper mine, fatty acid methyl esters, 16S rRNA, polyphasic taxonomy, *Saccharibacillus*

## Abstract

Gram-stain-positive, endospore-producing, mesophilic and rod-shaped strain O16^T^ was isolated from a copper mine’s soil and characterized using a polyphasic taxonomic approach. The 16S rRNA gene-sequence analysis revealed that strain O16^T^ belongs to the genus *Saccharibacillus*. It exhibited the highest sequence similarity to *Saccharibacillus endophyticus* JM-1350^T^ (97.2%), followed by ‘*Saccharibacillus alkalitolerans*’ VR-M41^T^ (97.1%), *Saccharibacillus sacchari* GR21^T^ (96.8%), *Saccharibacillus kuerlensis* HR1^T^ (96.6%), and *Saccharibacillus deserti* WLJ055^T^ (95.7%). Genome-based comparisons revealed that the digital DNA–DNA hybridization (dDDH) and average nucleotide identity (ANI) values between strain O16^T^ and its closest relatives, *S. endophyticus* JM-1350^T^ and ‘*S. alkalitolerans*’ VR-M41^T^, were 21.3% and 22.3%, and 76.6% and 77.6%, respectively, which are well below the recommended thresholds for species delineation. The diagnostic diamino acid of the cell wall was *meso*-diaminopimelic acid. Phosphatidylglycerol and diphosphatidylglycerol were the major polar lipids in strain O16^T^. The predominant menaquinone was MK-7. The DNA G+C content was 53.4%. The major cellular fatty acids present were anteiso-C_15:0_ (60.8%), iso-C_16:0_ (9.5%) and C_16:1_ ω11*c* (7.4%). On the basis of phenotypic, chemotaxonomic, and genotypic evidence, strain O16^T^ is considered to represent a novel species within the genus *Saccharibacillus*. This data strongly supports the classification of the strain O16^T^ as a novel species in the genus *Saccharibacillus*, for which we propose the name *Saccharibacillus soli* sp. nov. strain O16^T^ (=CCM 8781^T^ = KCTC 33898^T^).

## 1. Introduction

The genus *Saccharibacillus* belongs to family *Paneibacillaceae*, and was proposed by Rivas et al. [1]. The cells of this genus are Gram-variable, facultatively anaerobic, motile and rod-shaped. They are oxidase negative, contain ante-iso-C_15:0_ as the major fatty acid and menaquinone-7 (MK-7) as the major respiratory menaquinone. At the time of writing, the genus comprises seven species: *Saccharibacillus sacchari* [1], which is isolated from the inner tissues of sugarcane; ‘*Saccharibacillus brassicae*’ [2], obtained from kimchi cabbage seeds; ‘*Saccharibacillus alkalitolerans*’ [3], obtained from an open-air vegetable and fruit market; *Saccharibacillus kuerlensis* [4], obtained from desert soil; *Saccharibacillus deserti* [5], obtained from desert soil; *Saccharibacillus endophyticus* [6], obtained from stem tissue of cotton; and *Saccharibacillus quingshengii* [7], obtained from lead–cadmium tailings. Collectively, members of the genus demonstrate potential biotechnological applications, including hydrolytic enzyme production (e.g., cellulases), antimicrobial activity, tolerance to heavy metals such as lead and cadmium, and adaptation to extreme environments such as desert ecosystems. In this study, the bacterial strain O16^T^ isolated from a soil sample collected from copper mine is described. The 16S rRNA gene-sequence comparison revealed that the isolate is a *Saccharibacillus*-like organism. The present work is carried out to determine the exact taxonomic position of the isolate by a polyphasic approach.

## 2. Materials and Methods

### 2.1. Isolation of Strain O16^T^ and Procurement of Reference Strain

In an attempt to isolate heavy metal-resistant bacteria, strain O16^T^ was recovered from a soil sample collected at a copper mine site in Khetri, Rajasthan, India. The isolate was obtained using the dilution plate method on tryptic soy agar (TSA) medium (HiMedia; Mumbai, India) incubated at 30 °C. For all the studies, the isolate was cultivated on TSA medium at 30 °C and preserved at –70 °C as glycerol stocks. Strain O16^T^ has been deposited in the Culture Collection of the Czech Collection of Microorganisms under accession number CCM 8781^T^ and in the Korean Collection for Type Cultures under accession number KCTC 33898^T^ (Supplementary Data-Figure S2). The reference-type strains *Saccharibacillus kuerlensis* HR1^T^ (KCTC 13182^T^), *Saccharibacillus deserti* WLJ055^T^ (KCTC 33693^T^), *Saccharibacillus sacchari* GR21^T^ (DSM 19268^T^), and *Saccharibacillus endophyticus* JM-1350^T^ (CCM 8702^T^) were obtained from the Korean Collection for Type Cultures (KCTC), Deutsche Sammlung von Mikroorganismen und Zellkulturen GmbH (DSMZ) and Czech Collection of Microorganisms (CCM), respectively, and ‘*Saccharibacillus alkalitolerans*’ VR-M41^T^ (KCTC 43183^T^) was obtained from the authors’ laboratory [3].

### 2.2. Morphological and Physiological Characterisation

Strain O16^T^ and other closely related organisms given above were grown on TSA and tested for polyphasic taxonomic characteristics according to the proposed minimal standards for describing new taxa of aerobic, endospore-forming bacteria [8]. Colony morphology was determined according to standard methods, as described by Murray et al. [9]. Gram stain reaction was performed using Gram Staining kit (HiMedia, Mumbai, India). Cell morphology and motility were studied under Olympus BX51 microscope and transmission electron microscope (JEM-2100, JEOL, Tokyo, Japan) after growing cells in TSB at 30 °C for 24 h. Endospore formation was checked by observation with a phase contrast microscope and malachite-green staining of isolate grown on TSA (supplemented with 5 mg/L of MnSO_4_) for a week [8]. Physiological tests like growth at different temperatures (4 °C, 10 °C, 15 °C, 20 °C, 25 °C, 30 °C, 37 °C, 42 °C and 50 °C) and NaCl (1.0%, 2.0%, 3.0%, 4.0%, 5.0%, 6.0% and 7.0% *w*/*v*) concentrations were investigated by growing the strain on basal TSA medium. For checking growth at pH between 5.0 and 11.0, tryptic soy broth (TSB; HiMedia, Mumbai, India) was adjusted to different pH with biological buffer system (NaHCO_3_/Na_2_CO_3_ for alkaline pH and 1.0 M HCl for acidic pH). Catalase, oxidase reactions, citrate utilization (using Simmons’ citrate agar), and decomposition of urea were checked, as explained by Cowan and Steel [10]. Hydrolysis of gelatine, casein, tyrosine, starch and tween 20, 40, 60, and 80; methyl red test; Voges–Proskauer test; indole production; hydrogen sulfide production; and citrate utilization and motility were determined, as mentioned by Smibert and Krieg [11]. Nitrate reduction was tested, as described by Lanyi [12]. Various sugars were tested for acid production on minimal medium by method described by Clark [13]. VITEK 2 GP cards were used with 24-h-old culture and incubated at 30 °C, according to the instructions of manufacturer (BCL card, bioMérieux, Craponne, France).

### 2.3. Chemotaxonomic Characterisation

For chemotaxonomic analysis, freeze-dried cells were prepared after growing the strain in TSB for 2 d at 30 °C. Standard procedure was followed to determine the isomer type of the diaminopimelic acid of the peptidoglycan layer, as described by Staneck and Roberts [14]. Menaquinones extraction and analysis were carried out, as per methods described by Minnikin et al. [15] and Kroppenstedt [16]. Extraction of polar lipids and thin-layer chromatography (TLC) (Kieselgel 60 F254; Merck KGaA, Darmstadt, Germany) run were performed by methods described by Kaur et al. [17]. For cellular fatty acid analysis, the cells were grown on TSA medium at 30 °C for 36 h and the analysis of fatty acid methyl ester was carried out with the Sherlock Microbial Identification System (MIDI, Newark, DE, USA), as described previously [18].

### 2.4. Genome Sequencing, Annotation and Comparison

Genomic DNA was extracted, and the 16S rRNA gene was amplified and sequenced. Phylogenetic analysis was performed, as described previously [19]. The 16S rRNA gene sequence of the strain O16^T^ was used for sequence similarity search using the EzBioCloud web server [20] and aligned using Mega version 12 [21]. To find out the evolutionary distance, phylogenetic trees were constructed using neighbor-joining, maximum parsimony and maximum-likelihood algorithms. The sequencing of draft genome of strain O16^T^ was carried out by Genotypic Technology Pvt Ltd., Bengaluru, India, as per the methods described by Kumar et al. [22] and assembled using CLC Bio Workbench v7.5.1 (CLC Bio, Aarhus, Denmark), and graphical circular map of the genome performed with CGview comparison tool [23]. Whole-genome-based taxonomic analysis was performed using the Type (Strain) Genome Server (TYGS) available at https://tygs.dsmz.de (accessed on 24 February 2026). For phylogenomic inference, genome sequences included in the analysis were subjected to pairwise comparisons using the Genome BLAST Distance Phylogeny (GBDP) approach with the algorithm “trimming” and distance formula d5 to calculate accurate intergenomic distances [24]. One hundred distance replicates were generated for each genome pair to ensure statistical robustness. The resulting distance matrix served as the basis for tree construction using FASTME 2.1.6.1 [25], with branch lengths scaled according to the GBDP distance formula d5. The values shown above the branches represent GBDP pseudo-bootstrap support values > 60% based on 100 replications, with an average branch support of 72.2%. The final phylogenomic tree was midpoint-rooted and regenerated with the iTOL tool v6 [26]. Genome relatedness between strain O16^T^ and its closely related species was evaluated by calculating the orthologous average nucleotide identity (ortho-ANI) using the Orthologous Average Nucleotide Identity Tool (OAT) [27]. Digital DNA–DNA hybridization (dDDH) values were estimated using the Genome-to-Genome Distance Calculator (GGDC 2.0) available at http://ggdc.dsmz.de/distcalc2.php (accessed on 15 September 2025). The contig files were uploaded to the GGDC web server for analysis. dDDH values were computed using Formula (2), which provides genome length-independent estimates and is recommended for comparisons involving incomplete or draft genomes [28]. Genome annotation was done using the Rapid Annotation using Subsystem Technology (RAST) tool kit (RASTtk) (Arlington, VT, USA) of Bacterial and Viral Bioinformatics Resource Center (BV-BRC) via established pipelines for identification of protein-coding genes (CDS), rRNAs, tRNAs, and other genomic features [29].

Functional annotation of strain O16^T^ and the reference strains was performed by predicting coding DNA sequences (CDSs) in their genomes using Prokka (v1.14.6) [30]. The resulting annotation files (.gff format) were used as input for the pan-genome analysis Pipeline (PGAP2, v1.0.8) to perform pan-genome analysis [31]. The generated presence–absence matrix was further processed using base R functions to determine genome-specific gene clusters. Pairwise shared gene clusters were identified by computing set intersections between gene sets of genome pairs and enumerating the shared clusters, while three-way shared clusters were identified through nested comparisons to detect clusters simultaneously present in selected genome triplets. The processed data, together with the presence–absence matrix, were subsequently analyzed in Python (v3.12.9). Using the pandas, numpy, matplotlib.pyplot, os, and matplotlib.patches libraries, a customized six-way Euler-type Venn diagram was generated to visualize unique, core, and pairwise/three-way shared gene clusters, thereby providing an integrated representation of the pan-genome structure [32].

### 2.5. Copper Tolerance and Phosphate Solubilisation Assay

Copper tolerance of stain O16^T^ was evaluated using a liquid culture growth assay, as described by Cox et al. 2022 [33]. A cell suspension of strain O16^T^ (10^8^ CFU mL^−1^) was prepared from a 24 h-old culture and inoculated into nutrient broth supplemented with different concentrations of Cu^2+^ (added as CuSO_4_·5H_2_O) at 0, 1.0, 2.0, 3.0, 4.0, 5.0, 6.0, and 7.0 mM. The cultures were incubated for 2 days, and copper tolerance was determined based on cell viability.

For qualitative assessment of inorganic phosphate (P) solubilization, strain O16^T^ was streaked onto Pikovskaya’s (PVK) agar plates and incubated to observe the formation of a clear halo zone around the colonies, indicating the extent of inorganic phosphate solubilization [34,35]. Protein-coding genes putatively associated with copper tolerance and inorganic phosphate solubilization were identified through analysis of the annotated genome.

## 3. Result and Discussion

### 3.1. Morphological and Physiological Characteristics

**Figure 1:**
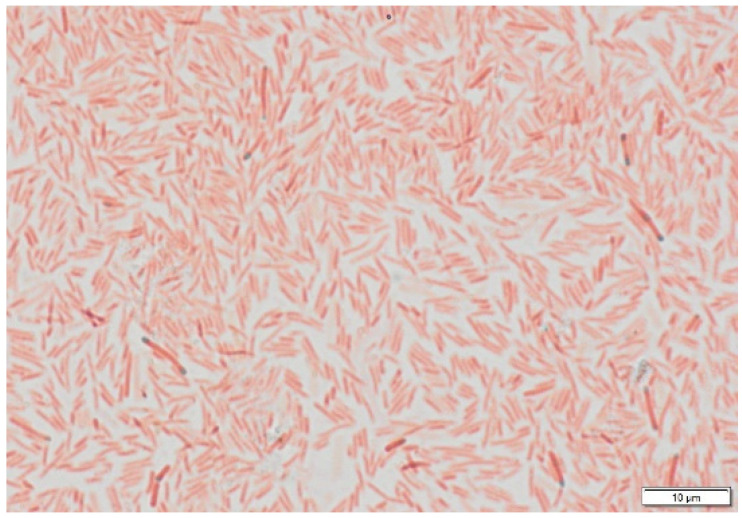
Microscopic image of strain O16^T^ showing terminal endospores, Bar 10 µm.

**Figure 2:**
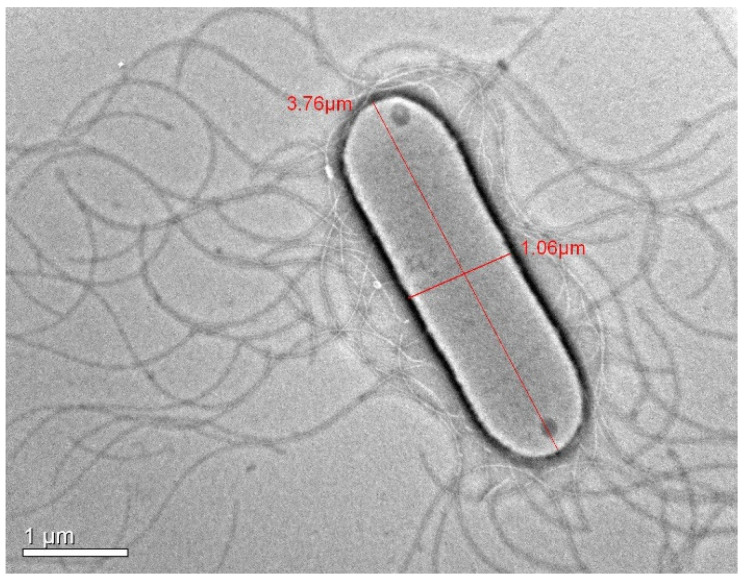
Transmission electron micrograph of strain O16^T^ showing peritrichous flagella, Bar 1 µm.

Cells are Gram-stain-positive, aerobic, endospore-forming, motile rods occurring singly or in pairs. Cells measure 3.5–4.7 μm in length and 0.8–1.0 μm in width (Figure 1 and Figure 2). Colonies on a TSA medium are orange-colored, opaque, round and convex with undulated margins. Endospores are terminal and round with bulging sporangia. They are positive for catalase and negative for oxidase tests. Growth occurred at 15–42 °C (optimum 30 °C) and at pH 6.0–11.0 (optimum pH 7.0). The strain tolerated up to 6.0% (*w*/*v*) NaCl, with a growth range of 0–6.0%. Strain O16^T^ produced acid from dextrose, sucrose, mannose, galactose, inulin, xylose, rhamnose, lactose, trehalose, melibiose, arabinose, mannitol, inositol, fructose, raffinose, cellobiose, salicin, and maltose. These reactions were largely consistent with those of the reference strains, except for rhamnose and inulin, for which acid production was absent in a few reference strains (Table 1). No acid production was observed from adonitol or dulcitol.

The strain was positive for nitrate reduction, methyl red test, and hydrolysis of tween 40 and tween 60, but negative for the Voges–Proskauer test, and for the hydrolysis of gelatin, casein, urea, and starch, as well as citrate utilization. In VITEK GP card analysis, positive reactions were observed for D-amygdalin, arginine dihydrolase 1 and 2, α-galactosidase, β-galactosidase, leucine arylamidase, β-galactopyranosidase, l-pyrrolidonyl arylamidase, alanine arylamidase, tyrosine arylamidase, D-galactose, lactose, D-maltose, D-mannose, D-mannitol, methyl-β-D-glucopyranoside, D-raffinose, sucrose, D-trehalose, and optochin resistance. Negative reactions were recorded for phosphatidylinositol phospholipase C, D-xylose, Ala-Phe-Pro arylamidase, cyclodextrin, l-aspartate arylamidase, α-mannosidase, phosphatase, l-proline arylamidase, β-glucuronidase, urease, D-sorbitol, D-ribose, polymyxin B resistance, l-lactate alkalinisation, N-acetyl-D-glucosamine, novobiocin resistance, bacitracin resistance, pullulan, and O/129 resistance.

All the strains were positive for growth at 10–37 °C, pH 6.0–8.0, and 0–4.0% (*w*/*v*) NaCl as well as for catalase activity, methyl red test and hydrolysis of tween 20, 40 and 60, whereas ‘*S. alkalitolerans*’ was negative for catalase activity and hydrolysis of these tweens. Growth at 7.0% (*w*/*v*) NaCl was not observed for any strain except ‘*S. alkalitolerans*’, which tolerated up to 8.0% NaCl. All strains were negative for indole production, citrate utilization, urease activity, the Voges–Proskauer test, and hydrolysis of starch, casein, gelatin, and tween 80. All strains were positive for arginine dihydrolase 1, arginine dihydrolase 2, and tyrosine arylamidase, as determined using the VITEK 2 GP card. The following reactions were negative for all strains in the VITEK 2 GP system: phosphatidylinositol phospholipase C, Ala–Phe–Pro arylamidase, cyclodextrin, L-aspartate arylamidase, α-mannosidase, phosphatase, L-proline arylamidase, β-glucuronidase, D-sorbitol, urease, polymyxin B resistance, N-acetyl-D-glucosamine, bacitracin resistance, novobiocin resistance, and pullulan, whereas ‘*S. alkalitolerans*’ showed a weakly positive reaction for D-sorbitol.

Differential characteristics of strain O16^T^, including its ability to grow at 42 °C, inability to tolerate pH 5.0, nitrate reduction, glucose fermentation pattern, inability to grow beyond 6.0% (*w*/*v*) NaCl, oxidase reaction, acid production from rhamnose and inulin, as well as differences in carbon-source utilization and enzyme activities, distinguish it from one or more closely related species. These phenotypic variations, considered within a polyphasic taxonomic framework, clearly separate strain O16^T^ from its phylogenetically neighboring species, *Saccharibacillus endophyticus* JM-1350^T^, ‘*S. alkalitolerans*’ VR-M41^T^, *S. sacchari* GR21^T^, *S. kuerlensis* HR1^T^, and *S. deserti* WLJ055^T^ (Table 1).

### 3.2. Chemotaxonomic Characteristics

**Figure 3:**
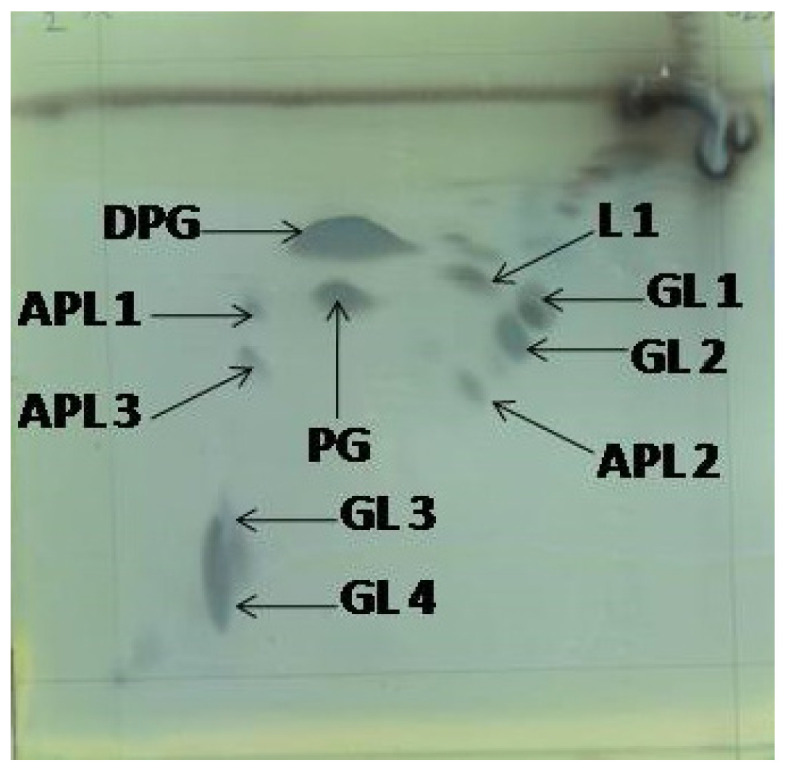
Polar lipid profile of O16^T^ determined by Two-dimensional TLC, detected by staining with molybdophosphoric acid (5% *w*/*v*) in absolute ethanol. Diphosphatidylglycerol (DPG), phosphatidylglycerol (PG), unidentified glycolipids (GL1–GL4), unidentified aminophospholipids (APL1–APL3), and unidentified polar lipid (L1).

The chemotaxonomic features of strain O16^T^ were consistent with its assignment to the genus *Saccharibacillus*. The diagnostic diamino acid of the cell-wall peptidoglycan was meso-diaminopimelic acid, which is characteristic of members of the genus and supports its placement within the family *Paenibacillaceae*. The predominant respiratory quinone detected was menaquinone MK-7, in agreement with the quinone system reported for recognized species of *Saccharibacillus*. The polar-lipid profile of strain O16^T^ comprised phosphatidylglycerol and diphosphatidylglycerol as the major components. In addition, three unidentified aminophospholipids, four unidentified glycolipids, and one unidentified polar lipid were detected (Figure 3; Supplementary Data-Figure S1). This polar-lipid composition is broadly comparable to that reported for other members of the genus, although minor differences in the unidentified lipid components may contribute to species-level differentiation. The major cellular fatty acids were anteiso-C_15:0_ (60.8%), iso-C_16:0_ (9.5%), and C_16:1_ ω11*c* (7.4%), with branched-chain fatty acids predominating (Table 2). The dominance of anteiso-C_15:0_ is a typical chemotaxonomic trait of the genus and supports its generic affiliation, while quantitative differences in fatty acid composition distinguish strain O16^T^ from its closest phylogenetic relatives.

### 3.3. Genome Analysis

**Figure 4:**
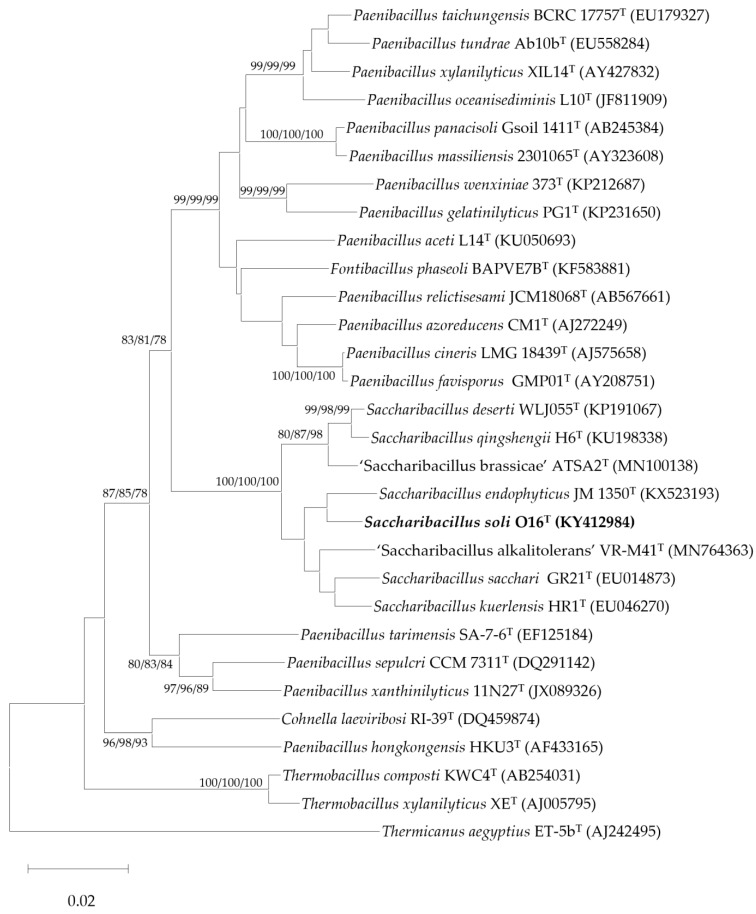
Phylogenetic neighbor-joining tree based on the 16S rRNA gene sequences showing the relationship between strain O16^T^ and other related members. *Thermicanus aegyptius* ET-5b^T^ (AJ242495) was used as an out-group. Bootstrap values > 70% were indicated at the branch points. Bar, 0.02 substitution for 100 nucleotide positions. The sequence accession numbers are given between parentheses. Bootstrap values ≥ 70% at branches are obtained for trees of neighbor-joining trees, minimum-evolution and maximum-likelihood, respectively.

**Figure 5:**
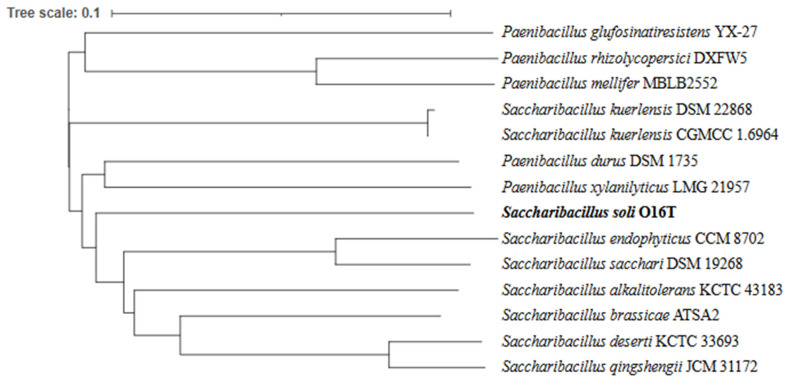
Whole-genome-based phylogenetic tree highlighting the taxonomic position of strain O16^T^ with its closely related species.

**Figure 6:**
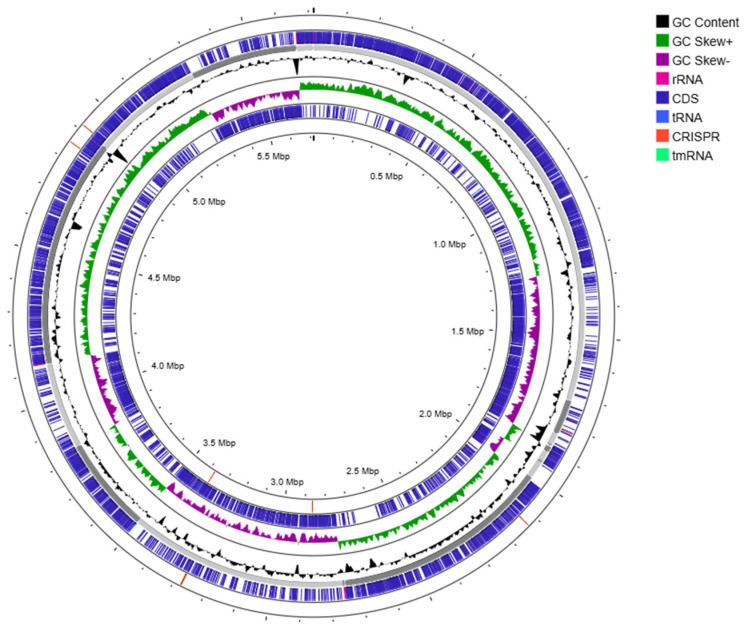
Genome graphical circular map of the strain O16^T^ obtained using the CGView server online tool.

Strain O16^T^ exhibited the highest 16S rRNA gene-sequence similarity to *Saccharibacillus endophyticus* JM-1350^T^ (97.2%), followed by ‘*S. alkalitolerans*’ VR-M41^T^ (97.1%), *S. sacchari* GR21^T^ (96.8%), *S. kuerlensis* HR1^T^ (96.7%), and *S. deserti* WLJ055^T^ (95.5%). The nearly complete 16S rRNA gene sequence (1505 bp) obtained in this study was aligned with sequences of other members of the genus *Saccharibacillus* retrieved from GenBank. Phylogenetic analysis demonstrated that strain O16^T^ formed a distinct lineage within the genus, clustering with *S. endophyticus*, ‘*S. alkalitolerans*’, *S. kuerlensis*, *S. sacchari*, and *S. deserti* (Figure 4). A phylogenomic tree based on the GTDB database highlights the taxonomic position of strain O16^T^ with its closely related species (Figure 5). Whole-genome relatedness was further assessed using average nucleotide identity (ANI) and digital DNA–DNA hybridization (dDDH) (Table 3). The dDDH values between strain O16^T^ and *S. endophyticus* JM-1350^T^, ‘*S. alkalitolerans*’ VR-M41^T^, *S. sacchari* GR21^T^, *S. kuerlensis* HR1^T^, and *S. deserti* WLJ055^T^ were 21.3%, 22.3%, 21.3%, 21.1%, and 21.6%, respectively. The corresponding ANI values were 76.6%, 77.6%, 76.6%, 76.3%, and 76.7%. These values are significantly below the accepted species delineation thresholds (70% for dDDH and 95–96% for ANI) [23,24,25], supporting the genomic distinctiveness of strain O16^T^. Illumina platform-based next-generation sequencing of strain O16^T^ generated a draft genome of 5,716,101 bp with ~200× coverage using 150 bp paired-end chemistry. The assembly showed 99.2% completeness, with no detectable contamination. The genome had a G+C content of 53.4% and comprised 25 contigs, 5177 coding DNA sequences (CDSs), five rRNA operons, and 58 tRNA genes, with an N50 value of 745,055 bp (Figure 6; Supplementary Data-Table S2). Genomic features of the reference strains are given in Table 4.

**Figure 7:**
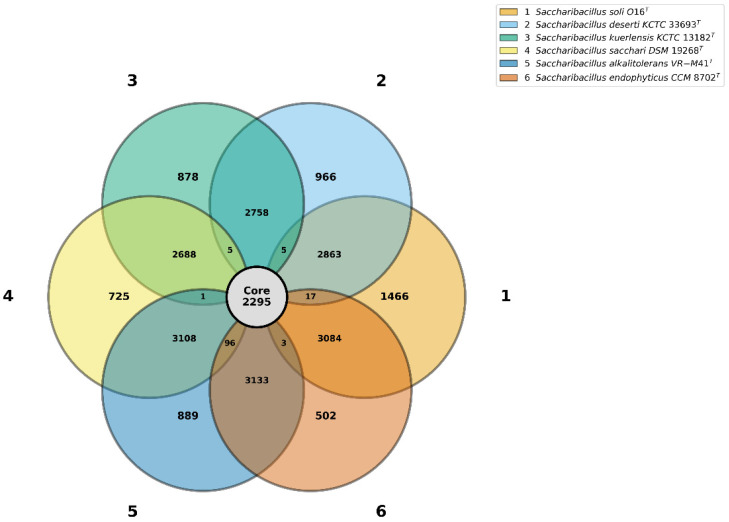
Venn diagram depicts the pan-genome analysis that represents the unique, accessory and core genes among strain O16^T^ (1) and its closely related species *S. deserti* WLJ055^T^ (2), *S. kuerlensis* HR1^T^ (3), *S. sacchari* GR21^T^ (4), ‘*S. alkalitolerans*’ VR-M41^T^ (5), and *S. endophyticus* JM-1350^T^ (6).

Pan-genome analysis of strain O16^T^ with *S. endophyticus* JM-1350^T^, ‘*S. alkalitolerans*’ VR-M41^T^, *S. sacchari* GR21^T^, *S. kuerlensis* HR1^T^, and *S. deserti* WLJ055^T^ identified 1466, 502, 889, 725, 878, and 966 unique genes, respectively, along with 2293 core genes shared among all strains. The respective accessory gene counts were 5972, 6333, 6341, 5898, 5457, and 5648 (Figure 7; Supplementary Data-Table S1). Notably, strain O16^T^ possessed a relatively higher number of unique genes, suggesting distinctive genetic features that may underlie its ecological adaptations and potential biotechnological applications.

### 3.4. Copper Tolerance and Phosphate Solubilisation Assay

To evaluate the potential application of the newly isolated strain O16^T^ as a plant-growth-promoting bacterium in heavy metal-contaminated soils, assays for copper tolerance and phosphate solubilization were performed, both of which yielded positive results. In parallel, genome analysis revealed the presence of protein-coding genes associated with these traits, including those related to copper tolerance (*CopZ*) and phosphate uptake/solubilization (*PstA*, *PstC*, and *PhnE*). The presence of these functional genes corroborates the phenotypic observations and supports the potential use of strain O16^T^ as a plant-growth-promoting bacterium in copper-contaminated environments.

Based on the combined genotypic and phenotypic evidence, strain O16^T^ can be clearly distinguished from its phylogenetic relatives. Accordingly, strain O16^T^ represents a novel species of the genus *Saccharibacillus*, for which the name *Saccharibacillus soli* sp. nov. is proposed.

### 3.5. Proposal of Saccharibacillus soli sp. nov.

*Saccharibacillus soli* (so′li. L. neut. gen. n. *soli* of soil, the isolation source of the type strain) cells are Gram-stain-positive, aerobic, endospore-forming, motile rods occurring singly or in pairs. Cells measure 3.5–4.7 μm in length and 0.8–1.0 μm in width. Colonies on a TSA medium are orange-colored, opaque, round and convex with undulated margins. Endospores are terminal and round with bulging sporangia. They are positive for catalase and negative for oxidase tests. Growth occurs at 15–42 °C (optimum 30 °C), between pH 6.0–11.0 (optimum 7.0) and NaCl ranges between 0 and 6.0% (optimum 3.0% *w*/*v*). Acid production is observed from dextrose, sucrose, mannose, galactose, inulin, xylose, rhamnose, lactose, trehalose, melibiose, arabinose, mannitol, inositol, fructose, raffinose, cellobiose, salicin and maltose; results are negative for adonitol and dulcitol. The strain is positive for nitrate reduction, methyl red test, and tween 40 and tween 60 hydrolysis; negative for Voges–Proskauer test, gelatine, casein, urea and starch hydrolysis, and citrate utilization; In VITEK GP cards were found positive for D-amygdalin, arginine dihydrolase 1, arginine dihydrolase 2, α-galactosidase, β-galactosidase, leucine arylamidase, β-galactopyranosidase, α-galactosidase, L-pyrrolidonyl-arylamidase, alanine arylamidase, tyrosine arylamidase, D-galactse, lactose, D-maltose, D-mannose, D-mannitol, methyl-B-D-glucopyranoside, D-raffinose, sucrose, D-trehalose, optochin resistance; negative for phosphatidylinisitol phospholipase C, D-xylose, Ala Phe Pro arylamidase, cyclodextrine, l-Aspartate arylamidase, α-mannosidase,phosphatise, l-proline arylamidase, β-glucoronidase, urease, D-sorbitol, D-ribose, polymyxin B resistance, l-lactate alkalinisation, N-acetyl-D-glucosamine, novobiocin resistance, bacitracin resistance, and pullulan and O/129 resistance. The diagnostic diaminoacid of the cell wall was *meso*-diaminopimelic acid. The major isoprenoid quinone is MK-7. Major polar lipids present are PG and DPG. Dominant fatty acids present are anteiso-C_15:0_ (60.8%), iso-C_16:0_ (9.5%) and C_16:1_ ω11*c* (7.4%).

The type strain is O16^T^ (=CCM 8781^T^ = KCTC 33898^T^), isolated from soil from Khetri copper mine site, Rajasthan, India.

## Figures and Tables

**Table 1:** Differentiating characteristics of strain O16^T^ and closely related species: O16^T^ (1), *S. endophyticus* JM-1350^T^ (2), ‘*S. alkalitolerans*’ VR-M41^T^ (3), *S. sacchari* GR21^T^ (4), *S. kuerlensis* HR1^T^ (5) and *S. deserti* WLJ055^T^ (6). w, weakly positive; +, positive; and -, negative.

Characteristics	1	2	3	4	5	6
Optimum temperature for growth (°C)	30	28	37	28	30	25
Growth at 42 °C	+	-	-	-	+	-
Growth at pH 5.0	-	+	-	+	-	+
NaCl tolerance % (*w*/*v*)	6.0	5.0	8.0	4.0	6.0	4.0
Nitrate reduction	+	+	-	+	-	-
Glucose fermentation	+	-	+	+	+	+
Oxidase	-	-	-	-	+	+
Acid production from:						
L-Rhamnose	+	+	w	-	-	-
Inulin	+	+	+	+	-	-
DNA G+C %	53.4	53.5	57.9	52.7	50.6	56.1
Results from VITEK 2 GP card						
D-amygdalin	+	-	+	+	+	+
D-xylose	-	-	+	+	+	+
beta-galactosidase	+	-	+	+	+	+
alpha-glucosidase	+	-	w	+	+	+
beta galactopyranosidase	+	-	+	+	+	+
leucine arylamidase	+	-	+	+	+	+
alpha galactosidase	+	-	+	+	+	+
L-pyrrolidonyl-arylamidase	-	-	-	+	+	+
alanine arylamidase	-	-	-	-	-	-
D-galactose	+	-	+	+	+	+
D-ribose	-	-	-	+	+	+
L-lactate alkalinization	-	+	+	+	+	+
lactose	+	-	w	+	+	+
D-maltose	+	-	+	+	+	+
D-mannitol	+	-	+	+	+	+
D-mannose	+	-	+	+	+	+
methyl-B-D-glucopyranoside	+	-	+	+	+	+
D-raffinose	+	-	+	+	+	+
salicin	+	-	+	+	+	+
saccharose/sucrose	+	-	+	+	+	+
D-trehalose	+	-	+	+	+	+

**Table 2:** Fatty acid component (%) profile of strain O16^T^ and closely related species: O16^T^ (1), *S. endophyticus* JM-1350^T^ (2), ‘*S. alkalitolerans*’ VR-M41^T^ (3), *S. sacchari* GR21^T^ (4), *S. kuerlensis* HR1^T^ (5) and *S. deserti* WLJ055^T^ (6). Summed feature 3 contained C_16:1_ ω6*c*/C_16:1_ ω7*c* and summed feature 4 contained C_18:2_ ω6, 9c/anteiso-C_18:0_; -, not detected.

Fatty Acid Type	1	2	3	4	5	6
C_12:0_	-	-	1.3	0.6	0.4	-
iso-C_13:0_ 3-OH	-	-	-	-	-	-
anteiso-C_13:0_	-	-	-	-	-	-
iso-C_14:0_	6.4	7.6	2.8	7.2	1.6	3.6
C_14:0_	1.7	1.6	1.4	1.6	0.6	0.7
iso-C_15:1_	-	-	-	-	-	-
anteiso-C_15:1_	-	-	-	-	-	-
iso-C_15:0_	4.7	2.5	1.7	2.1	2.4	2.5
anteiso-C_15:0_	60.8	54.3	42.2	58.0	55.5	64.9
C_16:1_ ω11*c*	7.4	-	5.2	-	4.7	-
C_16:1_ ω7*c* alcohol	1.4	-	2.0	-	6.3	0.6
iso-C_16:0_	9.5	6.3	4.1	10.2	3.5	9.1
C_16:0_	2.6	13.8	8.0	11.3	4.3	6.3
iso-C_17:1_ ω10*c*	-	-	1.0	-	1.6	-
iso-C_17:0_	0.6	-	1.7	1.7	1.0	1.3
anteiso-C_17:0_	2.8	2.1	11.7	6.5	7.2	9.2
C_17:0_	-	-	1.5	-	-	-
C_18:1_ ω9*c*	-	6.9	2	-	-	0.5
C_18:1_ ω5*c*	-	-	-	-	-	-
C_18:0_	1.0	1.5	4.7	-	-	0.6
Summed feature 3	-	1.3	1.8	-	0.4	-
Summed feature 4	0.5	2.2	-	-	9.3	-

**Table 3:** Comparative genomic data of ANI (top right) and dDDH (%) (bottom left) between strain O16^T^ (1) and its closely related species: *S. endophyticus* JM-1350^T^ (2), ‘*S. alkalitolerans*’ VR-M41^T^ (3), *S. sacchari* GR21^T^ (4), *S. kuerlensis* HR1^T^ (5), and *S. deserti* WLJ055^T^ (6).

	ANI						
**DDH**		**1**	**2**	**3**	**4**	**5**	**6**
	**1**		76.6	77.6	76.6	76.3	76.7
	**2**	21.3		79.4	79.3	79.2	80
	**3**	22.3	23.3		91.5	77.5	78.9
	**4**	21.3	23.1	44.7		77.4	91.5
	**5**	21.1	23	21.9	21.9		78.1
	**6**	21.6	23.7	22.8	22.8	22.2	

**Table 4:** Genomic features of strain O16^T^ and its closely related species.

Name of the Organism	Ref Sequence	Size (Mbp)	G+C (%)	CDS	RNA	tRNA	Protein Count
***Saccharibacillus soli* O16^T^**	NIOB00000000	5.7	53.4	5177	72	58	4910
***Saccharibacillus endophyticus* JM-1350^T^**	KX523193	5.9	53.5	5606	73	70	5097
***Saccharibacillus alkalitolerans* VR-M41^T^**	MN764363	5.3	57.9	5103	51	50	4563
***Saccharibacillus sacchari* GR21^T^**	NR 044375	6.0	52.7	5692	77	72	5263
***Saccharibacillus kuerlensis* HR1^T^**	NR 044389	4.6	50.6	4414	61	60	4078
***Saccharibacillus deserti* WLJ055^T^**	KP191067	5.1	56.1	4865	60	60	4455

## Data Availability

The data presented in this study are openly available in GenBank. The 16S rRNA gene sequence of Saccharibacillus soli strain O16T can be accessed under the accession number KY412984. The Whole Genome Shotgun project for strain O16T has been deposited at DDBJ/ENA/GenBank under the accession number NIOB00000000.

## Supplementary Materials

The following supporting information can be downloaded at: https://www.mdpi.com/article/10.3390/microorganisms14051150/s1, Figure S1. Polar lipid profile of strain O16^T^ determined by Two-dimensional TLC; Figure S2. Culture deposition certificates form Czech collection of microorganisms (CCM) and Korean collection for type culture (KCTC); Table S1. The pan-genome analysis that represents the unique genes, accessory genes, and core genes, of strain O16^T^ and its closely related species: *S. endophyticus* JM-1350^T^ (2), ‘*S. alkalitolerans*’ VR-M41^T^ (3), *S. sacchari* GR21^T^ (4), *S. kuerlensis* HR1^T^ (5), and *S. deserti* WLJ055^T^ (6); Table S2. Genome assembly quality parameters of strain O16^T^.
