# Supplementary material for: Taxonomic Description and Genomic Characterization of Saccharibacillus soli sp. nov., Isolated from Copper Mine Soil, Khetri, Rajasthan, India
Source: Microorganisms. 2026 May 19;14(5):1150. doi: 10.3390/microorganisms14051150 (PMC13209792; doi:10.3390/microorganisms14051150)
Supplement: Supplementary file 1 [file microorganisms-14-01150-s001.zip › microorganisms-4062622-supplementary.pdf]

Taxonomic Description and Genomic Characterization of *Saccharibacillus soli* Sp. nov., Isolated from copper Mine Soil, Khetri, Rajasthan, India

Himani[#] [1]; Bhawna Vyas[#] [1]; Munesh Kumari [1]; Ojal Bansal [4]; Shanmugam Mayilraj [1, 3]; Venkata Ramana Vemuluri [1,2*]

1. Microbial Type Culture Collection & Gene Bank (MTCC), CSIR-Institute of Microbial Technology (IMTECH), Sector 39-A, Chandigarh, 160 036, India.
2. Academy of Scientific and Innovative Research (AcSIR), Gaziabad 201002, India
3. Bentoli AgriNutrition India Pvt. Ltd., Anna Salai, Chennai 600 002, India
4. Department of Microbiology, Panjab University, South Campus, Sector-25, Chandigarh, 160014

* Correspondence: venkat.r@csir.res.in

[#]Equal authorship

Supplementary data- Figure S1. Polar lipid profile of strain O16T determined by two-dimensional TLC.

Detected by staining with molybdophosphoric acid (5% w/v) in absolute ethanol. Diphosphatidylglycerol (DPG), phosphatidylglycerol (PG), unidentified glycolipids (GL1-GL4), unidentified aminophospholipids (APL1-APL3), unidentified polar lipid (L1).

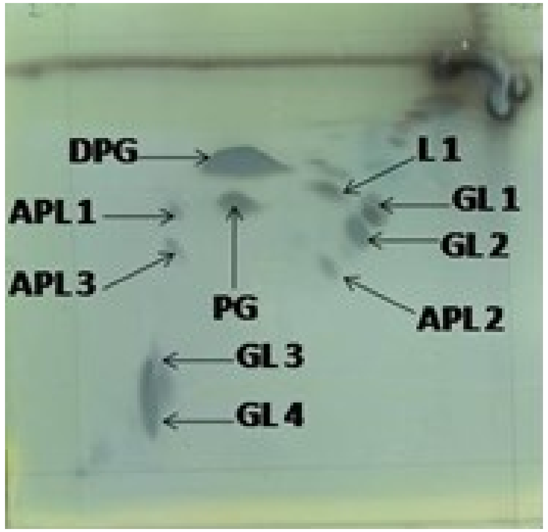

Total polar lipids

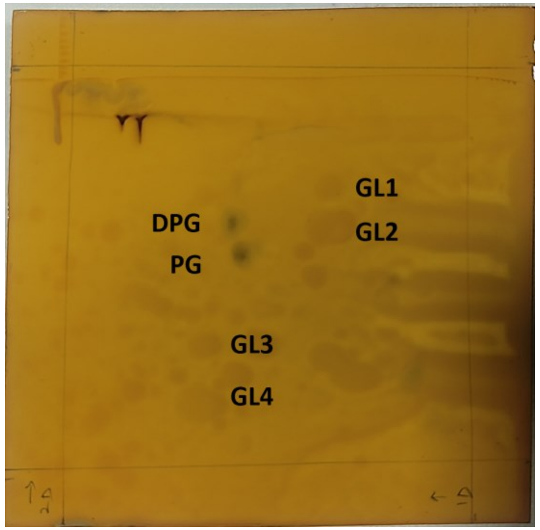

Staining of glycol and phospholipids

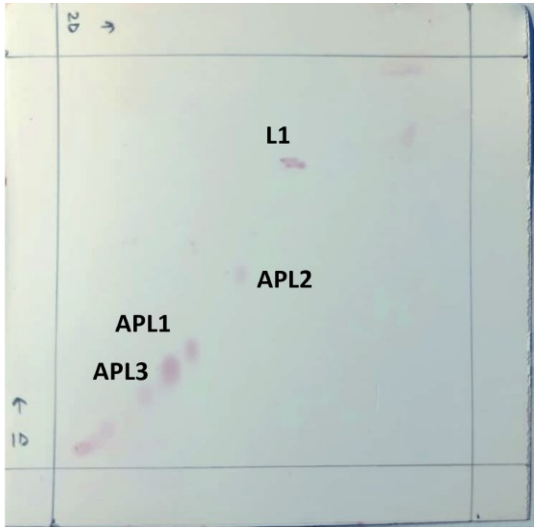

Staining of aminolipids

Supplementary data- Figure S2. Culture deposition certificates form Czech collection of microorganisms (CCM) and Korean collection for type culture (KCTC)

MASARYK UNIVERSITY
FACULTY OF SCIENCE
DEPARTMENT OF EXPERIMENTAL BIOLOGY

Czech Collection of Microorganisms (CCM)
tel.: +420 549 491 430 • fax +420 549 498 289
e-mail: ccm@sci.muni.cz • http://www.sci.muni.cz/ccm

Certificate of Deposit and Availability

We certify that the strain *Saccharibacillus soli* (O16) were received for deposition in the Czech Collection of Microorganisms (CCM) from

Dr. S. Mayilraj, from Microbial Type Culture Collection and Gene Bank (MTCC), CSIR-Institute of Microbial Technology, Chandigarh -160036, India

The strain was accessioned in the CCM as

CCM 8781 (=O16)

The strain will be available from the CCM public collection without any restriction after it will be validly published.

Restriction:

If the aforementioned strain and CCM accession number will not be published within 2 years since the deposition, CCM reserves the right to remove the strains from the collection.

Dana Nováková, Ph.D.
CCM Public Collection Curator

MASARYK UNIVERSITY
Faculty of Science
Czech Collection of Microorganisms
625 00 Brno, Kamenice 5
Czech Republic
VAT CZ00216224

Issued: **4/9/2017**
Valid up to: **4/9/2019**

IF-166.03 1/1

MASARYK UNIVERSITY, **FACULTY OF SCIENCE**, Kotlářská 2, 611 37 Brno, Czech Republic
Phone: +420 549 49 1411 • Fax: +420 541 211 214 • www.sci.muni.cz
VAT CZ00216224 • IBAN CZ43 0100 0000 0000 8563 6621

Korean Collection for Type Cultures (KCTC)
Korea Research Institute of Bioscience and Biotechnology (KRIBB)
181 Ipsin-gil, Jeongeup-si, Jeonbuk 56212, South Korea
Tel: +82-63-570-5602, FAX: +82-63-570-5609
E-mail: deposit@kribb.re.kr

Certificate of Deposit

Ref.: 20170301 Date of issue: APR 25, 2017

Taxonomic designation	:	***Saccharibacillus soli***
Accession number	:	KCTC 33898
Depositor(s)	:	Dr. Shanmugam Mayilraj
Strain code by the depositor(s)	:	**O16**

The above microorganism has been successfully deposited into the general collection of microorganism of the Korean Collection for Type Cultures (KCTC) and confirmed the identity of the microorganism under this KCTC number.
This microorganism will be available without restrictions for research and academic purposes in the publicly accessible section of the KCTC. It will be included in published and online catalogues after publication of this number by the authors.

Curator of Bacteria
Jung-Sook Lee Ph.D.

Telephone: +82-63-570-5618
Fax: +82-63-570-5609
E-mail: jslee@kribb.re.kr
Web: http://kctc.kribb.re.kr

Supplementary data- Table S1. The pan-genome analysis that represents the unique genes, accessory genes, and core genes, of strain O16^T and its closely related species: *S. endophyticus* JM-1350^T (2), '*S. alkalitolerans*' VR-M41^T (3), *S. sacchari* GR21^T (4), *S. kuerlensis* HR1^T (5), and *S. deserti* WLJ055^T (6).

**Genes**	**1**	**2**	**3**	**4**	**5**	**6**
Unique genes	1466	502	889	725	878	966
Accessory gene	5972	6333	6341	5898	5457	5648
Core genes	2295	2295	2295	2295	2295	2295

Supplementary data- Table S2. Genome assembly quality parameters of strain $O16^T$

S. No	Genome features	Details
1	Genome size	5.7 Mb
2	Contig number	25
3	N50	745055
4	Sequencing platform	Illumina
5	Read type	150bp chemistry
6	Coverage	200X
7	completeness	99.2%
8	Contamination estimates	0%
